# Recent advances in the understanding and management of bipolar disorder in adults

**DOI:** 10.12688/f1000research.12329.1

**Published:** 2017-11-21

**Authors:** Janusz K. Rybakowski

**Affiliations:** 1 Department of Adult Psychiatry, Poznan University of Medical Sciences, Poznan, Poland

**Keywords:** mood stabilizers, bipolar disorder, lithium, gene-environmental interaction

## Abstract

This article focuses on some aspects of recent progress in the neurobiology and treatment of bipolar disorder (BD) in adults. A molecular-genetic approach to the etiopathogenesis of the illness resulted in the findings of a genetic overlap between BD and other major psychiatric disorders. Furthermore, a poly-gene-environmental interaction in the development of the illness has been demonstrated. For the management of BD, new drugs with putative mood-stabilizing properties have been introduced in the past two decades. However, none of these can surpass lithium, the prototype mood-stabilizer, still considered the most specific drug for BD. Recent research on lithium, besides providing new data on the neurobiology of BD, has confirmed anti-suicidal, immunomodulatory, and neuroprotective properties of this drug.

## Introduction

The fifth edition of the *Diagnostic and Statistical Manual of Mental Disorders* (DSM-5) ^1^ classifies bipolar disorder (BD) as a separate category of mental illness. BD is a recurrent and often chronic condition that is generally characterized by episodes of mania, hypomania, depression, and mixed affective states. BD is estimated to have a worldwide prevalence rate of 2–5%, and the prevalence of type I BD (that is, BD with mania) is estimated at 1–2% and type II BD (that is, BD without mania) is estimated at 3–4% ^2^. BD causes significant suffering for patients and their families and has an estimated 10–20% lifetime suicide rate ^3^. Genetic predisposition for BD is high, the heritability index is estimated at 0.85 ^4^, and drugs with mood-stabilizing properties make the most important pharmacological modality in the treatment of BD ^5^.

This article aims to briefly review some of the latest advances in understanding the genetics of BD and in the use of mood stabilizers (MSs) in its treatment, with a special focus on lithium. Recent results of the molecular-genetic studies of BD show, on the one hand, a genetic overlap between BD and a number of psychiatric disorders and, on the other hand, the effect of epigenetic and environmental factors on the genetic predisposition to the illness. New drugs with putative mood-stabilizing properties, mainly atypical anti-psychotics, have been introduced in the past two decades. Also, new discoveries have appeared concerning lithium, the prototype of mood-stabilizing drugs, still considered the most specific for BD. Recent research using lithium as a research tool has provided new data on the neurobiology of BD and also provides evidence of the anti-suicidal, immunomodulatory, and neuroprotective properties of lithium.

## Genetic overlap of bipolar disorder

A significant role of genetic factors in the pathogenesis of BD was presented by Craddock and Sklar ^6^ in their review, published in 2013. In recent decades, previous findings from family and twin studies have been supported by linkage and genetic association research, using mainly the candidate gene approach. The latest evidence comes from the genome-wide association studies (GWASs) showing and replicating the association of BD with numerous gene polymorphisms such as CACNA1C, ODZ4, and NSAN. A polygenic contribution to the risk of illness has been postulated (that is, many risk alleles of small effect).

An important message coming from recent molecular-genetic research is that genetic susceptibility to BD can be shared with other psychiatric disorders. A report from the Cross-Disorder Group of the Psychiatric Genomic Consortium, after performing a GWAS for the five disorders—autism spectrum disorder, attention deficit hyperactivity disorder (ADHD), BD, major depressive disorder, and schizophrenia (SCH)—in 33,332 cases and 27,888 controls, showed that single-nucleotide polymorphisms (SNPs) at four loci (regions on chromosomes 3p21 and 10q24, SNPs within CACNA1C and CACNB2) attained genome-wide significance for several of these disorders ^7^.

The most relevant genetic overlap can occur between BD and SCH. In 2009, Lichtenstein *et al.* ^8^, searching more than 2 million nuclear families in Sweden, showed that first-degree relatives of probands with either SCH or BD were at increased risk of both these disorders. In the same year, the GWAS from the International Schizophrenia Consortium confirmed a substantial overlap of common polygenic variation between these two illnesses ^9^. It seems that the polygenic risk score of BD can influence the clinical dimension of mania in SCH while such a score of SCH can influence the clinical dimension of psychosis in BD ^10^. It was also found that a lesser contribution of very large copy number variations in BD, compared with SCH, makes a significant difference between two disorders ^11^.

For many years, clinicians have discussed diagnostic difficulties in differentiating BD with ADHD ^12^ as well as with borderline personality disorders ^13^. These problems were recently substantiated in GWASs. In the first of them, a significant genetic overlap between BD and ADHD was demonstrated ^14^, and in the second, an overlap of borderline personality disorder with BD, major depression, and SCH was reported ^15^.

O’Donovan and Owen ^16^ believe that the shared genetics of psychiatric disorders, demonstrated in numerous studies, points to extensive genic and allelic pleiotropy. According to them, the existence of risk alleles specific to a single diagnostic category, including BD, is unlikely.

## Gene-environmental interaction in bipolar disorder

An emerging paradigm for the pathogenesis of psychiatric disorders, including BD, has been a model of gene-environment interaction, linking the genome, epigenetic regulation, and environmental factors. Epigenetic mechanisms such as DNA methylation and histone modification can modulate gene expression in response to the environment, therefore exerting an effect on the pathophysiology and development of BD ^17, 18^. Epigenetic findings in BD were mostly obtained by means of peripheral blood and postmortem brain studies. In BD, abnormal DNA methylation can make a trait marker. Sugawara *et al.* ^19^ when studying monozygotic twins discordant for BD found promotor hypermethylation of the serotonin transporter gene in the twin with BD. The most prominent candidate gene for methylation studies in BD is the brain-derived neurotrophic factor ( *BDNF*) gene. Among patients with BD, the more pronounced *BDNF* exon I hypermethylation was found in type II compared with type I of the disorder ^20^. In a postmortem study of patients with BD, Rao *et al.* ^21^ found increased global histone H3 acetylation and hypermethylation of the promotor region for the drebrin-like protein gene. Interestingly, many abnormal epigenetic mechanisms, similar to the multiple polygenic variations mentioned above, can be comparable in patients with psychotic BD and SCH. For example, Dong *et al.* ^22^ showed increased DNA-methyltransferase1 binding to GABA-ergic and *BDNF* promoters in postmortem brain of both patients with BD and those with SCH. It seems that psychosis can also be a shared epigenetic trait.

Epidemiological studies have identified multiple environmental factors that can be associated with BD ^23, 24^. Many of these are akin to those operating in SCH and major depressive disorder. Among factors occurring during pregnancy, the most important could be an intrauterine infection with influenza. Canetta *et al.* ^25^ demonstrated that maternal serological influenza exposure was related to a fivefold greater risk of adult BD with psychotic features but not without such features, and this may suggest that prenatal influenza can be a risk factor for psychosis. The same group reported that the offspring exposed *in utero* to maternal smoking exhibited a twofold greater risk for BD in adulthood ^26^.

Among psychological factors associated with the onset and course of BD, childhood trauma and life events should be listed. Negative experiences in childhood, such as physical, sexual, and emotional abuse; physical and emotional neglect; and separation from parents, occur in patients with BD significantly more frequently than in the control population. In these patients, such events are associated with an earlier onset and more severe course of the illness, suicidal behavior, substance abuse, and somatic diseases ^27^. In adulthood, stressful life events can trigger manic or depressive episodes. A prospective study of 222 patients with BD shows that more than 60% of them experienced at least one life event 6 months before a new episode ^28^.

Seasonal and climate factors can also exert an effect on the onset and course of BD. Recently, an association of seasonal variation and BD symptoms was summarized in a systematic review by Geoffroy *et al.* ^29^. Also, a relationship between sunlight and the age of onset of BD was demonstrated in an international multisite study ^30^.

## Mood stabilizers

The concept of MSs as the main drugs used in BD treatment has evolved in the last half century. MSs can be defined as drugs that (1) reduce or ameliorate manic or depressive symptoms or both, (2) act prophylactically to prevent recurrent manic or depressive episodes or both, and (3) do not induce or worsen manic or depressive episodes.

In 1963, a British psychiatrist, Geoffrey Hartigan, published the first report describing the long-term mood-stabilizing properties of lithium carbonate ^31^. At the turn of the 1960s/1970s, other publications described possible mood-stabilizing effects of anti-convulsant drugs such as valproate ^32, 33^ and carbamazepine ^34, 35^. The first observation that the atypical anti-psychotic drug clozapine may exert MS effects was advanced in the 1990s ^36^. Subsequently, other atypical anti-psychotics (for example, olanzapine, quetiapine, aripiprazole, and risperidone) have been found to have MS properties ^37– 39^. A suggestion that the anti-convulsant lamotrigine can also have MS characteristics was proposed in 2002 ^40^. Because lithium and the classic anti-convulsant drugs preceded the introduction of newer MS agents by several decades, a proposal was made to group lithium, valproate, and carbamazepine as first-generation MSs and atypical anti-psychotics and lamotrigine as second-generation MSs ^37^.

Another classification of MSs can take into account the concept of “predominant polarity” (that is, manic or depressive) as the prevailing prophylactic effect of an MS ^40^. Clozapine, which exerts a predominant anti-manic and anti-psychotic action, might be placed at the extreme anti-manic end of the polarity continuum, being the MS with the greatest effect during mania (especially psychotic mania). Similarly, atypical anti-psychotics like olanzapine, aripiprazole, and risperidone may also be placed along the anti-manic end of the polarity continuum. While first-generation MSs like lithium, carbamazepine, and valproate also demonstrate greater anti-manic than anti-depressant activity, lithium appears to produce the greatest anti-depressant action. It appears that quetiapine may exert a balance of anti-manic and anti-depressant effectiveness and might best be considered to occupy the mid-position along the polarity continuum. Finally, lamotrigine may best be placed on the anti-depressant pole of the continuum ^41^. The use of both old and new MSs for the best therapeutic and prophylactic effect in BD has been elaborated in recent guidelines for BD treatment, such as those of the World Federation of Societies of Biological Psychiatry ^42^, the Canadian Network for Mood and Anxiety Treatments (CANMAT) ^43^, the British Association of Psychopharmacology ^44^, and the Collegium Internationale Neuropsychopharmacologicum (CINP) ^45^.

## Lithium – for bipolar disorder and beyond

Lithium, the first drug with reported mood-stabilizing properties ^31^, is still regarded as the first choice for prevention of mood episodes in BD. A recent meta-analysis of lithium’s prophylactic efficacy was performed by Severus *et al.* ^46^. A comparison of lithium with valproate in this respect made in the framework of the BALANCE (Bipolar Affective Disorder: Lithium/Anticonvulsant Evaluation) study showed a superiority of lithium ^47^. Lithium is also the drug with the longest history of use for mood stabilization in mood disorders. Recently, we described a series of BD patients receiving lithium continually for 40 or more years with excellent results ^48^.

In 1999, a Canadian psychiatrist, Paul Grof, introduced the term “excellent lithium responders” for patients responding to lithium monotherapy by not having further recurrences of the illness and thus able to live a totally normal life. He suggested that in such patients the illness runs a course with distinct affective episodes and periods of complete remission and have low psychiatric co-morbidity and frequent bipolar family history ^49^. This could reflect a “classic” form of the BDs, whose features are similar to those described by Kraepelin ^50^ as “manisch-depressives Irresein”. In our study on bipolar patients entering lithium prophylaxis in the 1970s (60 patients) and in the 1980s (49 patients), the percentages of patients not experiencing affecting episodes over a 10-year period were 35% and 27%, respectively, roughly one third of lithium-treated subjects ^51^.

The response to lithium can be regarded as an endophenotype of BD and therefore has been a subject of molecular-genetic studies. The review of the association between lithium prophylactic efficacy and the SNPs of numerous candidate genes was done by the author of this article ^52^. In 2008, the International Consortium of Lithium Genetic (ConLiGen) was founded with the aim of performing the GWAS of lithium response. Recently, the first results were published including 2,563 patients from 22 centers. The association between lithium response and a chromosome 21 region, containing two long, non-coding RNA genes, playing a role in gene expression regulation in the brain, was reported ^53^.

The response to lithium may also serve as a tool to elucidate the pathogenesis of BD. Among numerous studies performed in recent years, interesting results were obtained by using induced pluripotent stem cells (iPSCs) acquired from lithium-responsive patients with BD. In 2015, Mertens *et al.* ^54^ investigated the cellular phenotypes in hippocampal dentate gyrus-like neurons derived from iPSCs of patients with BD. In this model, the authors observed hyperactive action potential firing, which was selectively reversed by lithium only in neurons derived from patients who also responded to lithium treatment. The findings were confirmed by Stern *et al.* ^55^ in a similar model. They showed that neurons derived from patients with BD divide into intrinsically different subpopulations, according to patients’ lithium response. Recently, Tobe *et al.* ^56^, profiling the iPSC proteomics, showed that lithium alters the phosphorylation of collapsing response mediator protein-2 (CRMP2). We investigated the effect of long-term lithium treatment on very small embryonic-like stem cells (VSELs) and the mRNA expression of pluripotency and glial markers, in peripheral blood, in patients with BD. In patients not taking lithium, an increased number of VSELs, correlating with the duration of illness, and higher expression of markers were found. Long-term treatment with lithium reduced the number of VSELs and attenuated the expression of some markers, thus suppressing hyperactive regenerative and inflammatory processes occurring in the illness. The results may also show that VSELs in patients not treated with lithium provide a biological marker of the illness and its clinical progress ^57^.

Several decades of using lithium in BD have demonstrated some unique properties of this ion. In the early 1990s, we showed that long-term treatment with lithium can suppress labial herpes infections ^58^. Also, at that time, a multicenter study established that lithium can decrease mortality by reducing the occurrence of suicide ^59^. The anti-suicidal effect of lithium was fully confirmed in a recent meta-analysis ^60^, and lithium is currently regarded as a MS with the most pronounced anti-suicidal activity. Furthermore, intriguing results have been obtained in studies performed in Japan and Austria. They point to an inverse relationship between lithium concentration in drinking water and suicide rate in a given area ^61, 62^. This may suggest that even very low levels of lithium in drinking water play a role in reducing suicide risk within the general population.

A topic of great interest is a possible neuroprotective effect of lithium. Recent neuroimaging studies have indicated an increase in gray matter of prefrontal cortex and left cingulate in lithium-treated healthy persons and bipolar patients, mostly lithium responders ^63– 65^. Also, an increase of hippocampal volume of lithium-treated bipolar patients has been reported ^66, 67^. The biochemical underpinnings of lithium’s neuroprotective effect have been proposed, especially the inhibition by lithium of glycogen synthase kinase-3 (GSK-3) ^68^. Based on these findings, a postulate to use lithium as a therapeutic agent in neurodegenerative conditions such as Alzheimer’s disease (AD), Huntington’s disease (HD), multiple system atrophy (MSA), and amyotrophic lateral sclerosis (ALS) has been voiced in recent years ^69^. Data from large cohort studies suggest an association between lithium treatment and dementia risk reduction or reduced dementia severity ^70^. In a few clinical trials, lithium showed some therapeutic effect in amnestic mild cognitive impairment and AD, even with doses lower than those used for mood stabilization. On the other hand, studies of lithium in HD, MSA, and ALS were mostly negative ^71^.

## Conclusions

Recent research has brought about a paradigm change as to the role of genetic factors in the pathogenesis of BD, demonstrating a genetic overlap between bipolar and other major psychiatric disorders and a poly-gene-environmental interaction in the development of the illness. New drugs with MS properties have found their place in the treatment guidelines for BD. Recently, we have also witnessed a revival of interest in lithium as a tool for the elucidation of the pathogenesis of the illness and in its unique biological properties. Some of these properties (for example, neuroprotective) may prompt its use beyond psychiatry.

## References

[1] Diagnostic and Statistical Manual of Mental Disorders. Fifth edition (DSM-5). Arlington VA, American Psychiatric Association,2013 Reference Source

[2] Merinkangas KR Tohen M: Epidemiology of bipolar disorder in adults and children.In: Tsuang MT, Tohen MT, Jones PB (Eds), *Textbook in Psychiatric Epidemiology* John Wiley and Sons, Chichester, UK,2011;329–342. 10.1002/9780470976739.ch19

[3] Rihmer Z Dome P: Major mood disorders and suicidal behavior.In: *The International Handbook of Suicide Prevention*(eds. RC O'Connor & J Pirkis), John Wiley & Sons,2016;74–92. 10.1002/9781118903223.ch4

[4] McGuffin P Rijsdijk F Andrew M: The heritability of bipolar affective disorder and the genetic relationship to unipolar depression. *Arch Gen Psychiatry.* 2003;60(5):497–502. 10.1001/archpsyc.60.5.497 12742871

[5] Severus E Schaaff N Möller H: State of the art: treatment of bipolar disorders. *CNS Neurosci Ther.* 2012;18(3):214–8. 10.1111/j.1755-5949.2011.00258.x 22070572 PMC6493359

[6] Craddock N Sklar P: Genetics of bipolar disorder. *Lancet.* 2013;381(9878):1654–62. 10.1016/S0140-6736(13)60855-7 23663951

[7] Cross-Disorder Group of the Psychiatric Genomics Consortium: Identification of risk loci with shared effects on five major psychiatric disorders: a genome-wide analysis. *Lancet.* 2013;381(9875):1371–9. 10.1016/S0140-6736(12)62129-1 23453885 PMC3714010

[8] Lichtenstein P Yip BH Björk C: Common genetic determinants of schizophrenia and bipolar disorder in Swedish families: a population-based study. *Lancet.* 2009;373(9659):234–9. 10.1016/S0140-6736(09)60072-6 19150704 PMC3879718

[9] International Schizophrenia Consortium, Purcell SM Wray NR: Common polygenic variation contributes to risk of schizophrenia and bipolar disorder. *Nature.* 2009;460(7256):748–52. 10.1038/nature08185 19571811 PMC3912837

[10] Ruderfer DM Fanous AH Ripke S: Polygenic dissection of diagnosis and clinical dimensions of bipolar disorder and schizophrenia. *Mol Psychiatry.* 2014;19(9):1017–24. 10.1038/mp.2013.138 24280982 PMC4033708

[11] Green EK Rees E Walters JT: Copy number variation in bipolar disorder. *Mol Psychiatry.* 2016;21(1):89–93. 10.1038/mp.2014.174 25560756 PMC5038134

[12] Brus MJ Solanto MV Goldberg JF: Adult ADHD vs. bipolar disorder in the DSM-5 era: a challenging differentiation for clinicians. *J Psychiatr Pract.* 2014;20(6):428–37. 10.1097/01.pra.0000456591.20622.9e 25406047

[13] Frías Á, Baltasar I Birmaher B: Comorbidity between bipolar disorder and borderline personality disorder: Prevalence, explanatory theories, and clinical impact. *J Affect Disord.* 2016;202:210–9. 10.1016/j.jad.2016.05.048 27267293

[14] van Hulzen KJE Scholz CJ Franke B: Genetic Overlap Between Attention-Deficit/Hyperactivity Disorder and Bipolar Disorder: Evidence From Genome-wide Association Study Meta-analysis. *Biol Psychiatry.* 2017;82(9):634–41. 10.1016/j.biopsych.2016.08.040 27890468 PMC7027938

[15] Witt SH Streit F Jungkunz M: Genome-wide association study of borderline personality disorder reveals genetic overlap with bipolar disorder, major depression and schizophrenia. *Transl Psychiatry.* 2017;7(6):e1155. 10.1038/tp.2017.115 28632202 PMC5537640

[16] O'Donovan MC Owen MJ: The implications of the shared genetics of psychiatric disorders. *Nat Med.* 2016;22(11):1214–9. 10.1038/nm.4196 27783064

[17] Ludwig B Dwivedi Y: Dissecting bipolar disorder complexity through epigenomic approach. *Mol Psychiatry.* 2016;21(11):1490–8. 10.1038/mp.2016.123 27480490 PMC5071130

[18] Fries GR Li Q McAlpin B: The role of DNA methylation in the pathophysiology and treatment of bipolar disorder. *Neurosci Biobehav Rev.* 2016;68:474–88. 10.1016/j.neubiorev.2016.06.010 27328785 PMC5003658

[19] Sugawara H Iwamoto K Bundo M: Hypermethylation of serotonin transporter gene in bipolar disorder detected by epigenome analysis of discordant monozygotic twins. *Transl Psychiatry.* 2011;1:e24. 10.1038/tp.2011.26 22832526 PMC3309511

[20] D'Addario C Dell'Osso B Palazzo MC: Selective DNA methylation of BDNF promoter in bipolar disorder: differences among patients with BDI and BDII. *Neuropsychopharmacology.* 2012;37(7):1647–55. 10.1038/npp.2012.10 22353757 PMC3358733

[21] Rao JS Keleshian VL Klein S: Epigenetic modifications in frontal cortex from Alzheimer's disease and bipolar disorder patients. *Transl Psychiatry.* 2012;2(7):e132. 10.1038/tp.2012.55 22760556 PMC3410632

[22] Dong E Ruzicka WB Grayson DR: DNA-methyltransferase1 (DNMT1) binding to CpG rich GABAergic and BDNF promoters is increased in the brain of schizophrenia and bipolar disorder patients. *Schizophr Res.* 2015;167(1–3):35–41. 10.1016/j.schres.2014.10.030 25476119 PMC4451449

[23] Uher R: Gene-environment interactions in severe mental illness. *Front Psychiatry.* 2014;5:48. 10.3389/fpsyt.2014.00048 24860514 PMC4030208

[24] Aldinger F Schulze TG: Environmental factors, life events, and trauma in the course of bipolar disorder. *Psychiatry Clin Neurosci.* 2017;71(1):6–17. 10.1111/pcn.12433 27500795 PMC7167807

[25] Canetta SE Bao Y Co MD: Serological documentation of maternal influenza exposure and bipolar disorder in adult offspring. *Am J Psychiatry.* 2014;171(5):557–63. 10.1176/appi.ajp.2013.13070943 24480930 PMC4025955

[26] Talati A Bao Y Kaufman J: Maternal smoking during pregnancy and bipolar disorder in offspring. *Am J Psychiatry.* 2013;170(10):1178–85. 10.1176/appi.ajp.2013.12121500 24084820 PMC4086419

[27] Jaworska-Andryszewska P Rybakowski J: Negatywne doświadczenia dziecięce a powstawanie i przebieg choroby afektywnej dwubiegunowej. *Psychiatr Pol.* 2016;50(5):989–1000. 10.12740/PP/61159 27992891

[28] Simhandl C Radua J König B: The prevalence and effect of life events in 222 bipolar I and II patients: a prospective, naturalistic 4 year follow-up study. *J Affect Disord.* 2015;170:166–71. 10.1016/j.jad.2014.08.043 25240845

[29] Geoffroy PA Bellivier F Scott J: Seasonality and bipolar disorder: a systematic review, from admission rates to seasonality of symptoms. *J Affect Disord.* 2014;168:210–23. 10.1016/j.jad.2014.07.002 25063960

[30] Bauer M Glenn T Alda M: Relationship between sunlight and the age of onset of bipolar disorder: an international multisite study. *J Affect Disord.* 2014;167:104–11. 10.1016/j.jad.2014.05.032 24953482

[31] Hartigan GP: The Use Of Lithium Salts In Affective Disorders. *Br J Psychiatry.* 1963;109:810–4. 10.1192/bjp.109.463.810 14080575

[32] Lambert PA Carraz G Borselli S: [Neuropsychotropic action of a new anti-epileptic agent: depamide]. *Ann Med Psychol (Paris).* 1966;124(5):707–10. 5941463

[33] Lambert PA Borselli S Marcou G: [Long-term thymoregulative action of Depamide in manic-depressive psychoses]. *Ann Med Psychol (Paris).* 1971;2(3):442–8. 5128637

[34] Takezaki H Hanaoka M: The use of carbamazepine in the control of manic-depressive psychosis and other manic-depressive states. *Clin Psychiatry.* 1971;13:173–183.

[35] Okuma T Kishimoto A Inoue K: Anti-manic and prophylactic effects of carbamazepine (Tegretol) on manic depressive psychosis. A preliminary report. *Folia Psychiatr Neurol Jpn.* 1973;27(4):283–97. 10.1111/j.1440-1819.1973.tb02661.x 4801623

[36] Zarate CA Jr Tohen M Banov MD: Is clozapine a mood stabilizer? *J Clin Psychiatry.* 1995;56(3):108–12. 7883728

[37] Rybakowski JK: Aripiprazole joins the family of second-generation mood stabilizers. *J Clin Psychiatry.* 2008;69(5):862–3. 10.4088/JCP.v69n0522b 18681763

[38] Quiroz JA Yatham LN Palumbo JM: Risperidone long-acting injectable monotherapy in the maintenance treatment of bipolar I disorder. *Biol Psychiatry.* 2010;68(2):156–62. 10.1016/j.biopsych.2010.01.015 20227682

[39] Ketter TA Calabrese JR: Stabilization of mood from below versus above baseline in bipolar disorder: a new nomenclature. *J Clin Psychiatry.* 2002;63(2):146–51. 10.4088/JCP.v63n0210 11874216

[40] Colom F Vieta E Daban C: Clinical and therapeutic implications of predominant polarity in bipolar disorder. *J Affect Disord.* 2006;93(1–3):13–7. 10.1016/j.jad.2006.01.032 16650901

[41] Popovic D Reinares M Goikolea JM: Polarity index of pharmacological agents used for maintenance treatment of bipolar disorder. *Eur Neuropsychopharmacol.* 2012;22(5):339–46. 10.1016/j.euroneuro.2011.09.008 22000157

[42] Grunze H Vieta E Goodwin GM: The World Federation of Societies of Biological Psychiatry (WFSBP) guidelines for the biological treatment of bipolar disorders: update 2012 on the long-term treatment of bipolar disorder. *World J Biol Psychiatry.* 2013;14(3):154–219. 10.3109/15622975.2013.770551 23480132

[43] Yatham LN Kennedy SH Parikh SV: Canadian Network for Mood and Anxiety Treatments (CANMAT) and International Society for Bipolar Disorders (ISBD) collaborative update of CANMAT guidelines for the management of patients with bipolar disorder: update 2013. *Bipolar Disord.* 2013;15(1):1–44. 10.1111/bdi.12025 23237061

[44] Goodwin GM Haddad PM Ferrier IN: Evidence-based guidelines for treating bipolar disorder: Revised third edition recommendations from the British Association for Psychopharmacology. *J Psychopharmacol.* 2016;30(6):495–553. 10.1177/0269881116636545 26979387 PMC4922419

[45] Fountoulakis KN Grunze H Vieta E: The International College of Neuro-Psychopharmacology (CINP) Treatment Guidelines for Bipolar Disorder in Adults (CINP-BD-2017), Part 3: The Clinical Guidelines. *Int J Neuropsychopharmacol.* 2017;20(2):180–95. 10.1093/ijnp/pyw109 27941079 PMC5408976

[46] Severus E Taylor MJ Sauer C: Lithium for prevention of mood episodes in bipolar disorders: systematic review and meta-analysis. *Int J Bipolar Disord.* 2014;2:15. 10.1186/s40345-014-0015-8 25530932 PMC4272359

[47] BALANCE investigators and collaborators, Geddes JR Goodwin GM: Lithium plus valproate combination therapy versus monotherapy for relapse prevention in bipolar I disorder (BALANCE): a randomised open-label trial. *Lancet.* 2010;375(9712):385–95. 10.1016/S0140-6736(09)61828-6 20092882

[48] Permoda-Osip A Abramowicz M Kraszewska A: Kidney, thyroid and other organ functions after 40 years or more of lithium therapy: a case series of five patients. *Ther Adv Psychopharmacol.* 2016;6(4):277–82. 10.1177/2045125316643299 27536347 PMC4971597

[49] Grof P: Excellent lithium responders: people whose lives have been changed by lithium prophylaxis.In: Birch NJ, Gallicchio VS, Becker RW, eds. *Lithium: 50 Years of Psychopharmacology, New Perspectives in Biomedical and Clinical Research.*Cheshire, Connecticut, Weidner Publishing Group1999;36–51.

[50] Kraepelin E: Psychiatrie: Ein Lehrbuch für Studierende und Ärzte. Auflage. Barth, Leipzig,1899;6 Reference Source

[51] Rybakowski JK Chlopocka-Wozniak M Suwalska A: The prophylactic effect of long-term lithium administration in bipolar patients entering treatment in the 1970s and 1980s. *Bipolar Disord.* 2001;3(2):63–7. 10.1034/j.1399-5618.2001.030203.x 11333064

[52] Rybakowski JK: Genetic influences on response to mood stabilizers in bipolar disorder: current status of knowledge. *CNS Drugs.* 2013;27(3):165–73. 10.1007/s40263-013-0040-7 23378337 PMC3602611

[53] Hou L Heilbronner U Degenhardt F: Genetic variants associated with response to lithium treatment in bipolar disorder: a genome-wide association study. *Lancet.* 2016;387(10023):1085–93. 10.1016/S0140-6736(16)00143-4 26806518 PMC4814312

[54] Mertens J Wang QW Kim Y: Differential responses to lithium in hyperexcitable neurons from patients with bipolar disorder. *Nature.* 2015;527(7576):95–9. 10.1038/nature15526 26524527 PMC4742055

[55] Stern S Santos R Marchetto MC: Neurons derived from patients with bipolar disorder divide into intrinsically different sub-populations of neurons, predicting the patients' responsiveness to lithium. *Mol Psychiatry.* 2017. 10.1038/mp.2016.260 28242870 PMC5573640

[56] Tobe BTD Crain AM Winquist AM: Probing the lithium-response pathway in hiPSCs implicates the phosphoregulatory set-point for a cytoskeletal modulator in bipolar pathogenesis. *Proc Natl Acad Sci U S A.* 2017;114(22):E4462–E4471. 10.1073/pnas.1700111114 28500272 PMC5465887

[57] Ferensztajn-Rochowiak E Kucharska-Mazur J Tarnowski M: Stem cells, pluripotency and glial cell markers in peripheral blood of bipolar patients on long-term lithium treatment. *Prog Neuropsychopharmacol Biol Psychiatry.* 2018;80(Pt A):28–33. 10.1016/j.pnpbp.2017.06.013 28625858

[58] Rybakowski JK Amsterdam JD: Lithium prophylaxis and recurrent labial herpes infections. *Lithium.* 1991;2:43–47. Reference Source

[59] Müller-Oerlinghausen B Ahrens B Grof E: The effect of long-term lithium treatment on the mortality of patients with manic-depressive and schizoaffective illness. *Acta Psychiatr Scand.* 1992;86(3):218–22. 10.1111/j.1600-0447.1992.tb03255.x 1414416

[60] Cipriani A Hawton K Stockton S: Lithium in the prevention of suicide in mood disorders: updated systematic review and meta-analysis. *BMJ.* 2013;346:f3646. 10.1136/bmj.f3646 23814104

[61] Ohgami H Terao T Shiotsuki I: Lithium levels in drinking water and risk of suicide. *Br J Psychiatry.* 2009;194(5):464–5; discussion 446. 10.1192/bjp.bp.108.055798 19407280

[62] Kapusta ND Mossaheb N Etzersdorfer E: Lithium in drinking water and suicide mortality. *Br J Psychiatry.* 2011;198(5):346–50. 10.1192/bjp.bp.110.091041 21525518

[63] Monkul ES Matsuo K Nicoletti MA: Prefrontal gray matter increases in healthy individuals after lithium treatment: a voxel-based morphometry study. *Neurosci Lett.* 2007;429(1):7–11. 10.1016/j.neulet.2007.09.074 17996370 PMC2693231

[64] Bearden CE Thompson PM Dalwani M: Greater cortical gray matter density in lithium-treated patients with bipolar disorder. *Biol Psychiatry.* 2007;62(1):7–16. 10.1016/j.biopsych.2006.10.027 17240360 PMC3586797

[65] Moore GJ Cortese BM Glitz DA: A longitudinal study of the effects of lithium treatment on prefrontal and subgenual prefrontal gray matter volume in treatment-responsive bipolar disorder patients. *J Clin Psychiatry.* 2009;70(5):699–705. 10.4088/JCP.07m03745 19389332

[66] Yucel K McKinnon MC Taylor VH: Bilateral hippocampal volume increases after long-term lithium treatment in patients with bipolar disorder: a longitudinal MRI study. *Psychopharmacology (Berl).* 2007;195(3):357–67. 10.1007/s00213-007-0906-9 17705060

[67] Hajek T Bauer M Simhandl C: Neuroprotective effect of lithium on hippocampal volumes in bipolar disorder independent of long-term treatment response. *Psychol Med.* 2014;44(3):507–17. 10.1017/S0033291713001165 23721695

[68] Pasquali L Busceti CL Fulceri F: Intracellular pathways underlying the effects of lithium. *Behav Pharmacol.* 2010;21(5–6):473–92. 10.1097/FBP.0b013e32833da5da 20700048

[69] Vo TM Perry P Ellerby M: Is lithium a neuroprotective agent? *Ann Clin Psychiatry.* 2015;27(1):49–54. 25696782

[70] Donix M Bauer M: Population Studies of Association Between Lithium and Risk of Neurodegenerative Disorders. *Curr Alzheimer Res.* 2016;13(8):873–8. 10.2174/1567205013666160219112957 26892288

[71] Forlenza OV Aprahamian I de Paula VJ: Lithium, a Therapy for AD: Current Evidence from Clinical Trials of Neurodegenerative Disorders. *Curr Alzheimer Res.* 2016;13(8):879–86. 10.2174/1567205013666160219112854 26892289

